# Expression of DLK1 Gene in the Bone Marrow Cells of Patients with Myelodysplastic Syndromes and Its Clinical Significance

**DOI:** 10.7497/j.issn.2095-3941.2012.03.006

**Published:** 2012-09

**Authors:** Lan-zhu Yue, Rong Fu, Hua-quan Wang, Li-juan Li, Er-bao Ruan, Guo-jin Wang, Wen Qu, Yong Liang, Jing Guan, Yu-hong Wu, Hong Liu, Jia Song, Xiao-ming Wang, Li-min Xing, Zong-hong Shao

**Affiliations:** Department of Hematology, General Hospital of Tianjin Medical University, Tianjin 300052, China

**Keywords:** DLK1 gene, myelodysplastic syndromes, expression

## Abstract

**Objective:**

This study aims to investigate the expression of delta-like 1 (DLK1) gene in the bone marrow cells of patients with myelodysplastic syndromes (MDS) and to explore its molecular characteristics for the early diagnosis of MDS.

**Methods:**

The expression of DLK1 mRNA in the bone marrow cells of cases with MDS, acute myeloid leukemia (AML), and normal control groups were measured by real-time polymerase chain reaction and were analyzed for clinical significance.

**Results:**

Significantly higher expression of DLK1 mRNA was observed in the bone marrow cells of MDS patients (0.7342±0.3652) compared with the normal control group (0.4801±0.1759) (*P*<0.05). The expression of DLK1 mRNA had a positive correlation with the proportion of bone marrow blasts (*r*=0.467, *P*<0.05). Moreover, DLK1 mRNA expression was significantly increased as MDS progressed (*P*<0.05). Patients with abnormal karyotypes exhibited significantly higher expression of DLK1 mRNA (0.9007±0.4334) than those with normal karyotypes (0.6411±0.2630) (*P*<0.05). Subsequently, patients with highly expressed DLK1 (≥0.8) presented significantly higher malignant clone burden (0.4134±0.3999) than those with lower DLK1 expression (<0.8),(0.1517±0.3109), (*P*<0.05).

**Conclusions:**

The DLK1 gene was highly expressed in MDS patients, and was increased as MDS progressed. The expression of DLK1 mRNA was positively correlated with the proportion of the bone marrow blasts. A high expression of DLK1 gene suggested a higher malignant clone burden of MDS.

## Introduction

The myelodysplastic syndrome (MDS) involves a group of myeloid neoplasms that induce dysplasia and ineffective hematopoiesis in the bone marrow, which lead to cytopenia in one or more peripheral blood cell lineages. MDS has a high-risk of evolving into AML(acute myeloid leukemia)^[^^1^^]^. Abnormalities in the expression of several oncogenes and tumor suppressor genes were observed in MDS. However, no gene was found specific to MDS. Studies have shown that the DLK1 gene was highly expressed in several MDS patients, which promoted abnormal cell proliferation. Hence, in this study, the expression of DLK1 in MDS and AML patients was investigated, and its correlation with bone marrow blasts and malignant clone burden was further analyzed to determine a novel parameter specific for MDS diagnosis.

## Patients and Methods

### Patients

Thirty-six MDS patients diagnosed in Department of Hematology, General Hospital of Tianjin Medical University between 2009 and 2010 were studied. All patients were diagnosed according to WHO criteria ^[^^2^^]^. The MDS patients comprised 7 refractory anemia (RA), 5 refractory anemia with ringed sideroblasts (RARS), 14 refractory cytopenia with multi-lineage dysplasia (RCMD), 1 refractory anemia with excess blast-I (RAEB-I), and 9 RAEB-II patients. Among the MDS patients, 21 were male and 15 were female. The sex ratio was 1.4:1. The patients’ age ranged from 15 to 81 years, with a median age of 51.5 years. The expression of DLK1 gene was detected before the patients received any treatment.

Furthermore, 10 AML patients consisting of 3 cases with recurrent genetic abnormalities and 7 cases that were not otherwise specified were studied. Six of the AML patients were untreated or were relapsed cases, and the other 4 were in complete remission. Among the AML patients, 2 were male and 8 were female. The sex ratio was 0.25:1. The patients’ ages ranged from 14 to 72 years, with a median age of 46 years.Ten healthy persons were studied as the control group.

### Methods

The bone marrow mononuclear cells (BMMNC) of the AML patients and the control group were separated via Ficoll-Hypaque density gradient centrifugation. Total RNA was extracted from the BMMNC using the Trizol kit (Invitrogen, CA, USA). First-strand cDNA was synthesized from the 5 ug total RNA using the MMLV cDNA synthesis kit (Tianze biologicals Co. Ltd.) according to the manufacturer’s instructions. Polymerase chain reaction (PCR) was conducted in a 25 µL reaction mixture containing Taq polymerase, dNTP, forward and reverse primers, β-actin, and cDNA template. The primer sequences of DLK1 were 5’-CTG GAC GGT GGC CTC TAT GAA TG-3’ and 5’-ATC ATC CAC GCA GGT GCC TC-3’. The sequences of β-actin were 5’-CTA CAA TGA GCT GCG TGT GGC-3’ and 5’-CAG GTC CAG ACG CAG GAT GGC-3’. Initial denaturation was conducted for 5 min at 94°C, followed by 35 cycles for 45 s at 94°C, annealing for 50 s at 56°C, and then extension for 50 s at 72°C. PCR was terminated through a final extension for 7 min at 72°C. The PCR products were fractionated through 2% agarose gels and viewed under ultraviolet light after ethidium bromide staining. Semi-quantity analysis was performed using the gel documentation system.

### Statistical analysis

The SPSS 13.0 software was used for data measurement. The data were represented as mean±standard deviation (SD). Student’s *t*-test was used for mean comparisons. Spearman bivariate correlation analysis was used for correlation analysis. A *P*<0.05 indicated statistical significance.

## Results

### Expression of DLK1 gene in the bone marrow cells of MDS and AML patients

**Figure 1:**
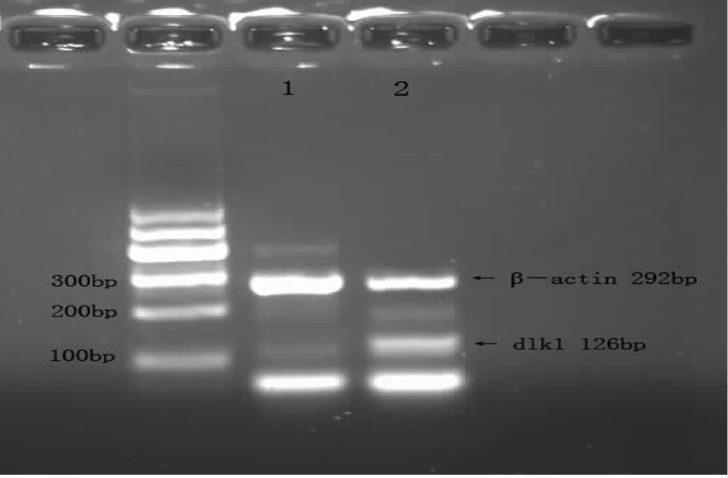
Expression of DLK1 gene in MDS and normal control groups. 1: Normal control group; 2: MDS patients

The expression of DLK1 mRNA in the bone marrow cells of MDS patients was significantly higher than that of the normal control group (*P*<0.05) (**Figure 1**). The expression of DLK1 gene in AML patients was higher than that of the normal control group, but lower than that of MDS patients, with no statistical significances (*P*>0.05). Furthermore, the level of DLK1 expression in the 6 untreated or relapsed AML patients was 0.6737±0.2748, which was lower than the MDS group but higher than the control group, with no significant differences (*P*>0.05, **Table 1**).

**Table 1:** Expression of DLK1 gene in MDS, AML, and normal control groups (mean±SD).

Group	Cases	Expression of DLK1
MDS	36	0.7342±0.3652*
AML	10	0.6402±0.2572
Normal control	10	0.4801±0.1759

* compared with the normal control group, *P*<0.05

### Expression of DLK1 gene in the MDS subtypes

The expression of DLK1 gene increased as MDS progressed from RA to RAEB. The level of DLK1 mRNA in the bone marrow cells of RAEB patients was significantly higher than in those of RA, RAS, and RCMD patients (*P*<0.05) (**Table 2**).

**Table 2:** Expression level of DLK1 gene in the MDS subtypes (mean±SD).

Group	Cases	Expression of DLK1
RA and RAS	12	0.5965±0.1968
RCMD and RCMD-RS	14	0.6385±0.2611
RAEB	10	1.0344±0.4830*

* compared with the other 2 groups, *P*<0.05

### Correlation between DLK1 expression and the proportion of blasts in the bone marrow cells of MDS

**Figure 2:**
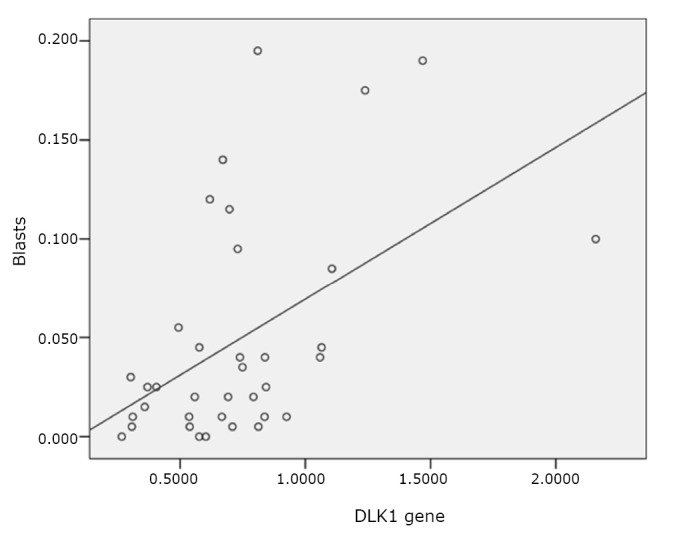
Correlation between DLK1 gene expression and bone marrow blasts proportion.

The expression of DLK1 mRNA was significantly correlated with the proportion of blasts in the bone marrow cells of MDS patients (*r*=0.467, *P*<0.05) (**Figure 2**). Twelve patients displayed a relatively higher expression of DLK1 (≥0.8), with an average proportion of bone marrow blasts of (7.67±7.23)%, whereas twenty-four patients had a relatively lower expression of DLK1 (<0.8), with a significantly lower proportion of bone marrow blasts [(3.52 ± 4.08)%] (*P*<0.05).

### Correlation between DLK1 gene expression and the number of cytopenic cell lineages in MDS

Seven patients displayed cytopenia in one cell lineage and eleven patients displayed cytopenia in two cell lineages. The average levels of DLK1 expression were (0.6465±0.1824) and (0.7458±0.3468), respectively. Eighteen patients had pancytopenia, with an average level of DLK1 expression at (0.7618±0.4337). As aforementioned, the expression of DLK1 mRNA became higher as the number of cytopenic cell lineages increased, but with no statistical differences (*P*>0.05).

### Correlations among DLK1 gene expression, karyotype and malignant clone burden in MDS

Chromosomal testing was conducted in 34 patients to determine their karyotypes. The results indicated that patients with abnormal karyotypes expressed significantly higher levels of DLK1 mRNA than those with normal karyotypes (*P*<0.05, **Table 3**).

**Table 3:** Correlation between expression of DLK1 and karyotypes.

Group	Cases	Expression of DLK1
Abnormal karyotypes	15	0.9007±0.4334*
Normal karyotypes	19	0.6411±0.2630

* compared with the normal karyotypes group, *P*<0.05

Subsequent analysis was performed on the correlation between DLK1 mRNA expression and malignant clone burden, which was designated as the ratio of the number of cells with abnormal karyotypes to the total mitotic cell counts (at least 20 mitotic figures were analyzed) in the bone marrow of MDS patients. Twelve and 22 patients displayed relatively higher and lower expressions of DLK1 (<0.8), respectively, with an average malignant clone burden of 0.4134±0.3999 and 0.1517±0.3109 (*P*<0.05), respectively.

### Correlation between DLK1 gene expression and IPSS

The International Prognostic Scoring System (IPSS) score was calculated according to the clinical data obtained from the MDS patients. The expression of DLK1 gene increased with increasing IPSS score, which was determined to be positively correlated with the IPSS score (*r*=0.449, *P*<0.05).

## Discussion

The DLK1 gene is a parent-derived imprinted gene located in the human chromosome 14q32. The DLK1 gene is widely expressed in various animals, ranging from birds to mammals ^[^^3^^]^. The DLK1 gene contains six exons sequences, which are highly conserved in humans, mice, and sheep. Furthermore, the DLK1 gene encodes a trans-membrane protein consisting of 383 amino acids, which is highly homologous with the structure of the mammalian Notch protein and belongs to the epidermal growth factor family^[^^4^^]^. The DLK1 protein is widely expressed in embryonic tissues. However, after birth, it selectively expresses in the pancreatic β cell, anterior pituitary, adrenal medulla, and testicular and ovarian cells. Moreover, DLK1 was found to be involved in embryonic development and hematopoiesis^[^^5^^]^. It is important in the differentiation and maturation of normal B cells because of its effect on the interaction between stroma cells and pre-B cells^[^^6^^]^.

Studies have shown that DLK1 gene is highly expressed in MDS patients^[^^7^^]^. Sakajiri et al.^[^^8^^]^ found that the expression of DLK1 mRNA in CD34+cells of MDS patients was significantly higher than that of the normal control group. High levels of DLK1 mRNA could be detected in BMMNCs of several MDS and leukemia patients. Moreover, several MDS-RA patients had elevated levels of DLK1 in plasma. Hence, these findings indicate the possibility of utilizing the DLK1 gene as a prospective parameter for the early diagnosis of MDS and for the determination of MDS pathogenesis.

In this study, we detected the expression of DLK1 mRNA in BMMNCs of MDS patients to investigate its expression level further, especially DLK1 levels in various MDS subtypes. We also wanted to determine its correlation with bone marrow blasts, karyotypes, and malignant clone burden, as well as to analyze its clinical significances. The results showed that DLK1 was highly expressed in MDS compared with the normal control group. As the MDS subtypes progress, the expression of DLK1 increased with the malignant clone burden and IPSS score. Thus, a high expression of DLK1 signifies a high malignant clone burden. The significant correlation of DLK1 expression with the proportion of bone marrow blasts indicates that DLK1 over-expression might promote hematopoietic stem cell proliferation. Moore et al.^[^^9^^]^ found that DLK1 transcription could be detected in stromal cell lines that support regenerating stem cells, but not in those without stem cell-supporting activity. This result indicates that DLK1 expression provided stromal cells with the ability to support proliferative stem cells. Furthermore, DLK1 gene might exert its effects by regulating the interaction between stromal cells and hematopoietic stem cells. In addition, several researchers found that DLK1 could possibly suppress cell differentiation, which might partially explain the differentiation abnormalities and ineffective hematopoiesis exhibited in MDS^[^^10^^−^^12^^]^.

Miyazato et al.^[^^13^^]^ investigated the gene expression profile of AC133+ cells of MDS and AML patients using oligonucleotide microarray. They found that DLK1 gene was specifically highly expressed in MDS. Most MDS patients presented high levels of DLK1 mRNA, with an average level higher than that of AML patients. In the 22 MDS patients that were studied, 12 presented DLK1 levels that were 2 times higher than that of the normal control group. On the other hand, out of the 31 AML patients that were studied, only 3 had a high expression of DLK1. Among these 3 patients, 1 displayed bone marrow dysplasia in 3 cell lineages, the second one presented 2 lineages dysplasia with P-H abnormality in the peripheral blood, and the third one had more than 98% blast proportion in the bone marrow, which made the observation of dysplasia difficult. Thus, 2 of the 3 AML patients were speculated to have probably evolved from MDS. These results showed that DLK1 gene might be specific for MDS, and that it can likely become a new diagnostic parameter of MDS.

For the mechanisms of DLK1, *in vitro* studies have shown that a high level of DLK1 could promote the proliferation of K562 cells probably by stimulating the transcription of HES1 and P21^WAF1^. DLK1 could suppress apoptosis induced by As_2_O_3_ and could affect cell cycles, which might contribute to the growth enhancement of malignant clones^[^^14^^]^. Other studies have shown that DLK1 gene could regulate the function of bone marrow mesenchymal cells by regulating and controlling the expression of several downstream genes. Thus, proliferation is promoted and the differentiation of hematopoietic cells is suppressed by altering the hematopoietic microenvironment^[^^15^^,^^16^^]^. In addition, synergistic effects were found between the signal pathways of DLK1 and Notch protein, which were consistent with their structural homology^[^^17^^−^^20^^]^. The Notch protein could promote hematopoietic cell proliferation by enhancing the expression of DLK1. Therefore, down-regulating DLK1 or blocking the Notch receptors could induce apoptosis and inhibit proliferation. A unique type of positive feedback might exist between the two pathways. Hence, mechanisms of DLK1 must be further investigated.

In conclusion, the expression of DLK1 gene was specifically higher in MDS patients and it increased as MDS subtypes progressed and bone marrow blasts increased. A high expression of DLK1 gene signifies a high malignant clone burden. Thus, DLK1 would probably be used as a new diagnostic parameter of MDS.
